# Observational and Genetic Associations of Body Mass Index and Hepatobiliary Diseases in a Relatively Lean Chinese Population

**DOI:** 10.1001/jamanetworkopen.2020.18721

**Published:** 2020-10-02

**Authors:** Yuanjie Pang, Christiana Kartsonaki, Jun Lv, Iona Y. Millwood, Canqing Yu, Yu Guo, Yiping Chen, Zheng Bian, Ling Yang, Junshi Chen, Robert Clarke, Robin Walters, Shukuan Wu, Huimei Li, Michael V. Holmes, Liming Li, Zhengming Chen

**Affiliations:** 1 Department of Epidemiology and Biostatistics, School of Public Health, Peking University, Beijing, China; 2 Clinical Trial Service Unit and Epidemiological Studies Unit, Nuffield Department of Population Health, Big Data Institute University of Oxford, Oxford, United Kingdom; 3 Medical Research Council Population Health Research Unit at the University of Oxford, Nuffield Department of Population Health, University of Oxford, Oxford, United Kingdom; 4 Chinese Academy of Medical Sciences, Beijing, China; 5 China National Center for Food Safety Risk Assessment, Beijing, China; 6 Haikou Meilan Disease Prevention and Control Center, Haikou, China; 7 National Institute for Health Research Oxford Biomedical Research Centre, Oxford University Hospitals National Health Service Foundation Trust, Oxford, United Kingdom

## Abstract

**Importance:**

There is some support for the existence of genetic associations between adiposity and certain hepatobiliary diseases in Western populations. However, there is little evidence of such genetic associations in China, where the causes of these diseases may differ from those in Western populations and the mean body mass index (BMI) is much lower.

**Objectives:**

To compare the observational associations of BMI with hepatobiliary diseases and liver biomarkers with the genetic associations between BMI and these factors and to assess whether the genetic associations of BMI with liver diseases differed by hepatitis B virus infection status.

**Design, Setting, and Participants:**

This cohort study used data from the prospective China Kadoorie Biobank, including 473 938 adults aged 30 to 79 years without hepatobiliary diseases at baseline from 10 diverse areas in China from June 25, 2004, to July 15, 2008. A random sample of 75 736 participants with genotyping data was included in the Mendelian randomization analysis. Follow-up was completed January 1, 2017 (median [interquartile range] length of follow-up, 10.2 [9.2-11.1] years). Data were analyzed from January to October 2019.

**Exposures:**

Measured BMI obtained during the baseline survey and genetically instrumented BMI derived using 92 single-nucleotide variations.

**Main Outcomes and Measures:**

Incident cases of hepatobiliary diseases, liver enzymes, fatty liver index, and fibrosis score.

**Results:**

Among 473 938 individuals (276 041 [58.2%] women), the mean (SD) age was 52 (10.9) years and mean (SD) BMI was 23.8 (3.4). Baseline BMI was associated with higher risks of chronic liver disease (adjusted risk ratio per 1-SD increase, 1.14; 95% CI, 1.11 to 1.17) and gallbladder disease (adjusted risk ratio per 1-SD increase, 1.29; 95% CI, 1.27 to 1.31), with heterogeneity by disease subtype (*P* < .001). Genetically instrumented BMI was associated with higher risks of chronic liver disease (risk ratio per 1-SD increase, 1.55; 95% CI, 1.08 to 2.24) and gallbladder disease (risk ratio per 1-SD increase, 1.40; 95% CI, 1.11 to 1.76), with no heterogeneity between subtypes. A meta-analysis of the genetic associations in China Kadoorie Biobank and those calculated in UK Biobank gave a risk ratio of 1.55 (95% CI, 1.30 to 1.84) for chronic liver disease and 1.42 (95% CI, 1.22 to 1.64) for gallbladder disease. In the China Kadoorie Biobank study, there were positive genetic associations of BMI with liver enzymes, steatosis, and fibrosis scores, consistent with observational associations. The genetic associations of BMI with liver diseases and biomarkers did not differ by hepatitis B virus infection status.

**Conclusions and Relevance:**

In this cohort study of a relatively lean Chinese population, there were positive genetic associations of BMI with hepatobiliary diseases. These results suggest that maintaining a healthy weight through diet and physical activity may help prevent hepatobiliary diseases.

## Key Points

**Question:**

Are there genetic associations of body mass index with hepatobiliary diseases in the Chinese population?

**Findings:**

In this cohort study including 473 938 adults, genetically instrumented body mass index was associated with higher risks of hepatobiliary diseases with different underlying causes and was associated with liver biomarkers, steatosis score, and fibrosis score. The genetic associations of body mass index with liver diseases and biomarkers were consistently observed regardless of hepatitis B virus infection.

**Meaning:**

These findings suggest that maintaining a healthy weight through diet and physical activity may help lower risk of hepatobiliary diseases in Chinese individuals regardless of the underlying causes of disease.

## Teaser

This cohort study compares observational associations with genetic associations of body mass index with hepatobiliary diseases and liver biomarkers.

## Introduction

Hepatobiliary disease is a major cause of morbidity and mortality worldwide, responsible for 2.4 million deaths in 2017, with approximately 20% of these occurring in China.^1^ In Western populations, alcohol consumption is the major cause of liver disease, associated with 30% to 60% of liver disease diagnoses,^2,3,4^ whereas hepatitis C virus accounts for 20% to 30% of liver disease in the West.^2^ In China, by contrast, hepatitis B virus (HBV) accounts for 50% and alcohol consumption accounts for approximately 10% of liver disease.^5,6^ Gallstones affect 10% to 20% of the global adult population^7,8^ and account for at least 90% of gallbladder disease in Western populations and 40% to 60% of the disease in Chinese populations.^8,9^

Prospective cohort studies in Western populations^10,11^ have consistently demonstrated positive associations of adiposity, mostly identified via body mass index (BMI; calculated as weight in kilograms divided by height in meters squared), with chronic liver disease and gallbladder disease. In contrast, a 2019 study in China^12^ reported that associations with BMI differed by chronic liver disease subtype, with a positive association for nonalcoholic fatty liver disease, an inverse association for liver cancer, and a U-shaped association for cirrhosis. A 2015 meta-analysis^11^ examined symptomatic gallbladder disease and did not differentiate between disease subtypes (ie, cholelithiasis vs cholecystitis). Previous studies in Western populations^13,14^ have reported some genetic evidence for the associations of BMI with risks of chronic liver disease and symptomatic gallbladder disease. However, previous genetic studies have been constrained by the small number of individuals the studies included, which meant they lacked power to reliably assess the associations by disease subtypes.

There is limited evidence for the association of BMI and these diseases in populations in East Asia, where mean BMI and the causes of hepatobiliary diseases differ from those in Western populations. Moreover, there is limited evidence for the genetic associations of BMI with biomarkers, including those associated with liver function, steatosis, and fibrosis. Additional evidence for these associations may provide insights into the underlying mechanisms. Understanding the genetic associations of BMI with hepatobiliary diseases may improve understanding of disease causes in diverse populations and inform prevention strategies.^15,16^

We present findings from the China Kadoorie Biobank (CKB) study population of approximately 500 000 adults. The objectives of this study were to compare the observational associations between BMI and hepatobiliary diseases and liver biomarkers with the genetic associations between BMI and these factors and to assess whether the genetic associations of BMI with liver diseases differed by HBV infection status.

## Methods

### Study Population

Details of the CKB prospective cohort study design, survey methods, and population characteristics have been described elsewhere.^17^ Briefly, 512 715 participants aged 30 to 79 years were recruited from 10 (ie, 5 urban and 5 rural) diverse areas in China from 2004 to 2008. All participants provided written informed consent. Prior international, national, and regional ethical approvals were obtained; this study was approved by the ethical committee and research council of the Chinese Center for Disease Control and Prevention and the Oxford Tropical Research Ethics Committee at the University of Oxford. At local study assessment clinics, each participant completed an interviewer-administered, laptop-based questionnaire on sociodemographic characteristics, smoking history, alcohol consumption, diet, physical activity, personal and family medical history, and current medication. Trained technicians recorded physical measurements (ie, height, weight, hip circumference, waist circumference, bio-impedance, lung function, blood pressure, and heart rate) using calibrated instruments and standard protocols. In addition, desktop analyzers were used to measure random blood glucose and hepatitis B surface antigen (HBsAg) (Acon Biotech). Trained technicians recorded all anthropometric measurements while participants wore light clothes and no shoes, with measurements taken usually to the nearest 0.1 cm or 0.1 kg. Standing height was measured using a stadiometer. Weight was measured using a body composition analyzer (TBF-300GS, Tanita), with clothing weight subtracted according to season (ranging from 0.5 kg in summer to 2.0-2.5 kg in winter). The baseline survey included quality control measures and assessment of liver biomarkers (eAppendix in the Supplement).

### Follow-up for Mortality and Morbidity

The vital status of each participant was determined periodically through the Chinese Center for Disease Control and Prevention’s Disease Surveillance Points system,^18^ supplemented by regular checks against local residential records and health insurance records and by annual active confirmation via street committees or village administrators. Additional information about major diseases and any episodes of hospitalization was collected by linkage, via each participant’s unique national identification number, to disease registries (for cancer, ischemic heart disease, stroke, and diabetes) and national health insurance claims databases (for hepatobiliary diseases). The databases had almost universal coverage in the study areas. Trained staff who were blinded to baseline information^18^ coded all events using *International Statistical Classification of Diseases and Related Health Problems, Tenth Revision (ICD-10)*.^19^ In the classification of hepatobiliary diseases, nonalcoholic fatty liver disease and alcoholic liver disease were classified entirely using medical records (eTable 1 in the Supplement). A total of 44 066 participants (9%) died and 4751 participants (<1%) were lost to follow-up by January 1, 2017.

### Genotyping and Biochemistry Measurements

Genotyping was conducted using a custom-designed 800K–single-nucleotide variation (SNV) (formerly single-nucleotide polymorphism [SNP]) array (Axiom, Affymetrix) with imputation to 1000 Genomes Phase 3. Genotype data were available for 100 408 participants whose samples passed quality control (ie, overall call rate >99.97% across all variants). Participants with genotype data consisted of a population-based sample of 75 736 participants randomly selected from the total CKB cohort and 24 672 participants who had been selected for nested case-control studies of incident stroke, coronary heart disease, or chronic obstructive pulmonary disease (eFigure 1 in the Supplement). To avoid selection bias, only the 75 736 randomly selected participants were used for genetic analyses of hepatobiliary outcomes. Among genotyped participants who had been selected for nested case-control studies of stroke and coronary heart disease, 17 567 participants were assayed for biomarkers associated with liver function (ie, aspartate transaminase [AST], alanine aminotransferase [ALT], and γ-glutamyl transferase) at the Nuffield Department of Population Health Wolfson Laboratories at the University of Oxford using baseline plasma samples (eAppendix in the Supplement).

### Observational Analyses

This study excluded 38 775 individuals with a history of hepatobiliary disease and 2 individuals with missing BMI, leaving 473 938 individuals in the observational analyses. Cox proportional hazard models were used to estimate hazard ratios of specific disease incidence associated with per 1-SD higher baseline BMI, adjusted for age (at baseline), education level, smoking history, and alcohol consumption, with stratification by sex, study area (from among 10 areas), and HBsAg (for chronic liver disease).

### Single-Nucleotide Variation Selection and Construction of Genetic Score

We used an unweighted genetic score for predisposition to higher BMI as an instrumental variable. The BMI genetic score was derived by summing up the number of BMI-increasing alleles using 97 independent (*r*^2^ ≤ 0.01 in the CEU [Centre d’Etude du Polymorphism Humain – Utah residents with Northern and Western European ancestry] population) BMI SNVs reported in a genome-wide association study by the GIANT (Genetic Investigation of Anthropometric Traits) consortium.^20^ Two variants (ie, rs12016871 and rs2245368) were not available in CKB, and 3 variants (ie, rs13107325, rs17024393, and rs2121279) were excluded because of the low minor allele frequency in the East Asian population (<1%), leaving 92 SNVs for the BMI genetic score. Per-allele effect sizes for BMI in CKB were estimated for the individual genetic variants in the GIANT consortium (eTable 2 in the Supplement). The BMI genetic score has been shown to perform well (*F* statistic, 1071; variance explained, 1.1%) and was not associated with potential confounders (eFigure 2 in the Supplement).

### Mendelian Randomization

The genetic associations of BMI with hepatobiliary diseases were calculated by the 2-stage least-squares estimator method using individual participant–level data. In the first stage, the associations between BMI genetic score and BMI were examined in 75 736 participants in the genome-wide association study population subset using linear regression, adjusting for age, age squared, sex, region, the first 12 principal components, education level, smoking history, and alcohol consumption. In the second stage, the associations of the resulting estimated values with hepatobiliary diseases were examined using logistic regression, adjusting for the same covariates plus HBsAg (for chronic liver disease). We calculated the genetic estimates per increase in genetically predicted BMI, equivalent to 1 SD baseline BMI, on disease outcomes to compare with associations with measured BMI.

### Statistical Analysis

The genetic estimates were compared with the corresponding observational estimates using a Cochran *Q* test. Odds ratios were used to approximate relative risks (RRs) of hepatobiliary diseases because of the rarity of events in CKB (<1.5%). Risk ratios and hazard ratios were used interchangeably. Therefore, we used the term RRs to refer to odds ratios and hazard ratios for consistency between the observational and genetic analyses. Statistical analyses included liver biomarker analysis, sensitivity analyses, and meta-analysis (eAppendix in the Supplement). The statistical analysis was performed January 2019 to October 2019 using R statistical software version 2.14.2 (R Project for Statistical Computing). *P* values are from Wald tests, and *P* < .05 was considered significant.

## Results

Of 473 938 participants, mean (SD) BMI was 23.8 and mean (SD) age was 52 (10.9) years; 276 041 (58.2%) were women. During 10 years of follow-up, there were 5904 cases of chronic liver disease and 16 720 cases of gallbladder disease. We ascertained all nonalcoholic fatty liver disease diagnoses between 2013 and 2015 and found that 1033 of 1111 individuals hospitalized for the disease (93%) were diagnosed by ultrasonography or computed tomography. Participants with chronic liver disease were more likely to be men, whereas participants with gallbladder disease were more likely to be women (Table 1). Participants with chronic liver disease and gallbladder disease were older and more likely to live in rural areas, have lower levels of education, and have lower household income. Participants with chronic liver disease, particularly men, were more likely to smoke (2396 of 3296 men [72.7%]; 89 of 2608 women [3.4%]) and consume alcohol weekly (1289 of 3296 men [39.1%]; 60 of 2608 women [2.3%]); participants with chronic liver disease were also more likely have test results positive for HBsAg (1222 of 5904 participants [20.7%]). Participants with chronic liver disease and gallbladder disease had higher mean levels in measures of systolic blood pressure, random plasma glucose, BMI, and waist circumference and were more likely to have prevalent diabetes and hypertension. No differences in female reproductive factors were observed across disease status.

**Table 1.** Baseline Characteristics of Participants by Incident Disease Status

Characteristic^a^	Without disease (n = 451 314)	Chronic liver disease (n = 5904)	Gallbladder disease (n = 16 720)
Age, mean (SD), y	51.7 (10.7)	56.0 (10.4)	53.3 (10.2)
Women, No. (%)	262 961 (58.0)	2608 (40.9)	10 472 (70.7)
Socioeconomic and lifestyle factors			
Urban region, No. (%)	201 494 (44.4)	2142 (37.3)	3021 (20.5)
≥9 y of education, No. (%)	96 243 (20.9)	1028 (20.1)	1529 (20.0)
Household income ≥35 000 renminbi^b^/y, No. (%)	80 224 (17.6)	907 (16.6)	2423 (17.1)
Ever regular smoking, No. (%)			
Men	128 892 (67.8)	2349 (72.7)	2980 (66.7)
Women	6792 (2.7)	169 (3.4)	462 (3.1)
Weekly alcohol consumption, No. (%)			
Men	63 939 (33.6)	1267 (39.1)	1288 (31.9)
Women	5319 (2.0)	84 (2.3)	265 (2.5)
Total physical activity (SD), MET-h/d	21.2 (13.8)	20.2 (13.6)	20.8 (14.1)
Sedentary leisure time (SD), h/d	3.0 (1.5)	3.0 (1.6)	3.0 (1.6)
Blood pressure and anthropometry, mean (SD)			
Systolic blood pressure, mm Hg	131.1 (21.3)	132.6 (21.9)	131.8 (21.6)
Random plasma glucose, mg/dL	110.0 (41.4)	115.3 (54.1)	111.7(43.2)
BMI categories, No. (%)			
Within reference range (≥18.5 to <25)	286 837 (63.4)	3472 (60.2)	8551 (54.7)
Overweight (25 to <30)	128 745 (28.3)	1832 (29.5)	4859 (35.4)
Obese (≥30)	17 866 (3.9)	316 (5.6)	787 (6.7)
BMI, mean (SD)	23.6 (3.4)	23.9 (3.6)	24.4 (3.5)
Waist circumference, mean (SD), cm	80.1 (9.7)	81.2 (10.1)	82.6 (10.0)
Hip circumference, mean (SD), cm	90.9 (6.8)	91.1 (7.3)	92.1 (6.9)
Waist-to-hip ratio, mean (SD)	0.90 (0.07)	0.90 (0.07)	0.90 (0.07)
Body fat, mean (SD), %	27.8 (8.3)	27.9 (9.2)	29.5 (8.7)
Height, mean (SD), cm	158.8 (8.3)	158.8 (8.5)	158.9 (8.0)
Female reproductive factors			
Age at menarche, mean (SD), y	15.4 (2.0)	15.5 (2.1)	15.4 (2.0)
Menopause, No. (%)	133 586 (51.2)	1741 (54.1)	6185 (52.4)
Pregnancies, mean (SD), No.	3.3 (1.7)	3.3 (1.9)	3.4 (1.7)
Live births, mean (SD), No.	2.2 (1.3)	2.3 (1.5)	2.3 (1.4)
With HBsAg, No. (%)	11 529 (2.6)	1161 (20.7)	311 (3.1)
Prior disease, No (%)			
Diabetes	25 552 (5.6)	539 (9.1)	935 (7.0)
Coronary heart disease	12 599 (2.8)	179 (2.6)	401 (3.3)
Stroke or TIA	7803 (1.7)	103 (1.4)	187 (1.7)
Hypertension	50 886 (11.3)	863 (13.1)	1911 (12.8)
Family history of disease, No. (%)			
Diabetes	22 306 (13.8)	227 (15.0)	480 (14.7)
Cancer	62 911 (4.9)	815 (4.4)	1663 (5.5)

Abbreviations: BMI, body mass index (calculated as weight in kilograms divided by height in meters squared); HBsAg, hepatitis B surface antigen; MET, metabolic equivalent of task; TIA, transient ischemic attack.

SI conversion factor: To convert random plasma glucose to millimoles per liter, multiply by 0.0555.

^a^ Results were standardized by age, sex, and region (where appropriate).

^b^ Approximately US $5080.

### Observational and Genetic Associations of BMI With Hepatobiliary Diseases

There were positive associations of BMI with risks of chronic liver disease and gallbladder disease, and the association was log-linear in the range 15 to 50 for gallbladder disease and the range 24 to 50 for chronic liver disease (eFigure 3 in the Supplement). The adjusted RRs per 1-SD increase were 1.14 (95% CI, 1.11 to 1.17) for chronic liver disease and 1.29 (95% CI, 1.27 to 1.31) for gallbladder disease (Table 2). Among disease subtypes, there was heterogeneity for chronic liver disease and gallbladder disease (*P* for heterogeneity < .001). For the observational association of chronic liver disease subtypes, the RR per 1-SD increased BMI was 0.69 (95% CI, 0.59 to 0.80) for alcoholic liver disease, 2.00 (95% CI, 1.90 to 2.10) for nonalcoholic fatty liver disease, 0.99 (95% CI, 0.95 to 1.04) for cirrhosis, 1.03 (95% CI, 0.97 to 1.09) for viral hepatitis, and 0.95 (95% CI, 0.91 to 0.99) for liver cancer. For the observational association of gallbladder disease subtypes, the RR per 1-SD increased BMI was 1.43 (95% CI, 1.41 to 1.46) for gallstone disease and 1.17 (95% CI, 1.15 to 1.20) for cholecystitis (Table 2).

**Table 2.** Observational and Genetic Associations of BMI With Risk of Hepatobiliary Diseases

Disease^a^	RR per 1-SD greater BMI (95% CI)	*P* for heterogeneity
**Chronic liver disease**		
Alcoholic liver disease		
Observational	0.69 (0.59-0.80)	.44
Genetic	1.59 (0.19-13.40)	
Nonalcoholic fatty liver disease		
Observational	2.00 (1.90-2.10)	.84
Genetic	1.79 (0.61-5.24)	
Cirrhosis		
Observational	0.99 (0.95-1.04)	.18
Genetic	1.76 (0.77-4.06)	
Viral hepatitis		
Observational	1.03 (0.97-1.09)	.23
Genetic	1.92 (0.70-5.26)	
Liver cancer		
Observational	0.95 (0.91-0.99)	.06
Genetic	1.70 (0.93-3.10)	
Overall		
Observational	1.14 (1.11-1.17)	.10
Genetic	1.55 (1.08-2.24)	
**Gallbladder disease**		
Gallstone disease		
Observational	1.43 (1.41-1.46)	.46
Genetic	1.62 (1.17-2.24)	
Cholecystitis		
Observational	1.17 (1.15-1.20)	.80
Genetic	1.20 (1.01-1.45)	
Overall		
Observational	1.29 (1.27-1.31)	.51
Genetic	1.40 (1.11-1.76)	

Abbreviations: BMI, body mass index; RR, relative risk.

^a^ The model was adjusted for age at baseline, age squared, 10 regions, the first 12 principal components (for genetic associations), hepatitis B surface antigen (for chronic liver disease), education level, smoking history, alcohol consumption, and total physical activity.

Among chronic liver diseases, there was a U-shaped association for cirrhosis and liver cancer, whereas there was a positive log-linear association for other chronic liver diseases (ie, hospitalized alcoholic liver disease, nonalcoholic fatty liver disease, and viral hepatitis) (eFigure 3 in the Supplement). For gallbladder disease, BMI was positively associated with cholelithiasis (RR per 1-SD increase, 1.43; 95% CI, 1.41 to 1.46) and cholecystitis (RR per 1-SD increase, 1.17; 95% CI, 1.15 to 1.20) (eFigure 3 in the Supplement).

Among individuals who were never regular smokers or when excluding the first 5 years of follow-up, there was no association of BMI with risk of cirrhosis in the BMI range 15 to 24, and there was a positive association in the BMI range 24 to 50; similarly for liver cancer, there was no association with BMI in the BMI range 15 to 24, and there was a positive association in the BMI range 24 to 50 (eFigure 6 in the Supplement).

For BMI and chronic liver disease, the genetic association (RR per 1-SD increase, 1.55; 95% CI, 1.08 to 2.24) did not differ from the observational association (RR per 1-SD increase, 1.14; 95% CI, 1.11 to 1.17) (*P* = .10) (Table 2). The BMI score was positively associated with chronic liver disease, with an RR of 1.55 (95% CI, 1.08 to 2.24) per 1-SD greater genetically instrumented BMI. Positive genetic associations were observed across chronic liver disease subtypes, with no heterogeneity (*P* for heterogeneity = .99). For the genetic association of chronic liver disease subtypes with BMI, the RR per 1-SD increase in BMI was 1.59 (95% CI, 0.19 to 13.40) for alcoholic liver disease, 1.79 (95% CI, 0.61 to 5.24) for nonalcoholic fatty liver disease, 1.76 (95% CI, 0.77 to 4.06) for cirrhosis, 1.92 (95% CI, 0.70 to 5.26) for viral hepatitis, and 1.70 (95% CI, 0.93 to 3.10) for liver cancer (Table 2).

Likewise, the genetic association of BMI with gallbladder disease did not differ from the observational association (Table 2), with an RR of 1.40 (95% CI, 1.11 to 1.76) per 1-SD greater genetically instrumented BMI and an RR of 1.29 (95% CI, 1.27 to 1.31) per 1-SD greater observational BMI. The genetic association of BMI with gallbladder disease did not differ by disease subtype (*P* for heterogeneity = .12). The RR per 1-SD greater genetically instrumented BMI was 1.62 (95% CI, 1.17 to 2.24) for cholelithiasis and 1.20 (95% CI, 1.01 to 1.45) for cholecystitis.

### Observational and Genetic Associations of BMI With Liver Biomarkers

For liver enzymes, there were positive associations of BMI with ALT (SD difference per 1-SD greater BMI, 0.16; 95% CI, 0.15 to 0.18) and γ-glutamyl transferase (SD difference per 1-SD greater BMI, 0.07; 95% CI, 0.06 to 0.08), whereas there was no association with AST (SD difference per 1-SD greater BMI, 0.01; 95% CI, −0.01 to 0.02) (Table 3). There were also positive associations of BMI with fatty liver index (SD difference per 1-SD greater BMI, 0.75; 95% CI, 0.74 to 0.76) and BARD (BMI, AST/ALT ratio, and diabetes) score (SD difference per 1-SD greater BMI, 0.12; 95% CI, 0.11 to 0.13). The genetic associations of BMI with liver biomarkers were consistent with observational associations, with the exception of BARD score (Table 3).

**Table 3.** Observational and Genetic Associations of BMI With Liver Biomarkers

Liver biomarker^a^	SD difference per 1-SD greater BMI (95% CI)	*P* for heterogeneity
**ALT, per 1-SD increase**		
Observational	0.16 (0.15 to 0.18)	.44
Genetic	0.12 (0.02 to 0.23)	
**AST, per 1-SD increase**		
Observational	<0.01 (−0.01 to 0.02)	.91
Genetic	<0.01 (−0.09 to 0.09)	
**γ-glutamyl transferase, per 1-SD increase**		
Observational	0.07 (0.06 to 0.08)	.17
Genetic	0.12 (0.04 to 0.20)	
**Fatty liver index, per 1-SD increase**		
Observational	0.75 (0.74 to 0.76)	.92
Genetic	0.75 (0.64 to 0.86)	
**BARD, per 1-unit**		
Observational	0.12 (0.11 to 0.13)	.001
Genetic	0.19 (0.15 to 0.22)	

Abbreviations: ALT, alanine aminotransferase; AST, aspartate transaminase; BMI, body mass index; BARD, BMI, AST/ALT ratio, and diabetes score.

^a^ The model was adjusted for age at baseline, age squared, 10 regions, the first 12 principal components (for genetic associations), hepatitis B surface antigen, education level, smoking history, alcohol consumption, total physical activity, and fasting hours.

### Subgroup and Sensitivity Analyses

**Figure 1.**
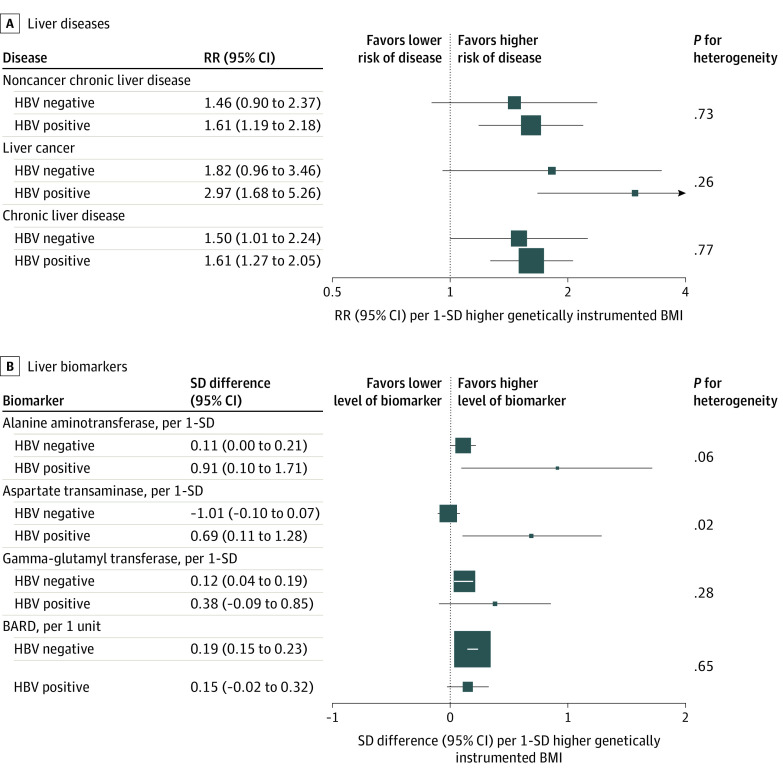
Genetic Associations With Body Mass Index by Hepatitis B Virus (HBV) Status A. Boxes indicate the relative risks (RRs) of liver diseases associated with 1-SD greater genetically determined body mass index (BMI; calculated as weight in kilograms divided by height in meters squared) in participants stratified by HBV status, with the size of the box inversely proportional to the variance of the log RR. B. Boxes indicate the SD differences of liver biomarkers associated with 1-SD greater genetically determined body mass index (BMI) in participants stratified by HBV status, with the size of the box inversely proportional to the variance of the SD difference. The genetic analysis was adjusted for age, age squared, sex, region, the first 12 principal components, education level, smoking history, and alcohol consumption. *P* values for comparison were obtained from a Cochran *Q* test comparing the observational and genetic estimates.

The positive genetic associations of BMI with risks of chronic liver disease were consistent in participants regardless of HBV status. For noncancer chronic liver disease, RR per 1-SD increase was 1.46 (95% CI, 0.90 to 2.37) among participants without HBV infection and 1.61 (95% CI, 1.19 to 2.18) among participants with HBV infection (*P* for heterogeneity = .73); for liver cancer, RR per 1-SD increase was 1.82 (95% CI, 0.96 to 3.46) among participants without HBV infection and 2.97 (95% CI, 1.68 to 5.26) among participants with HBV infection (*P* for heterogeneity = .26); and for chronic liver disease, RR per 1-SD increase was 1.50 (95% CI, 1.01 to 2.24) among participants without HBV infection and 1.61 (95% CI, 1.27 to 2.05) among participants with HBV infection (*P* for heterogeneity = .77) (Figure 1).

Similarly, there were positive genetic associations by HBV status for 3 liver biomarkers. For ALT, SD difference was 0.11 (95% CI, 0 to 0.21) among participants without HBV infection and 0.91 (95% CI, 0.10 to 1.71) for participants with HBV infection (*P* for heterogeneity = .06); for γ-glutamyl transferase, SD difference was 0.12 (95% CI, 0.04 to 0.19) among participants without HBV infection and 0.38 (95% CI, −0.09 to 0.85) among participants with HBV infection (*P* for heterogeneity = .28); and for BARD score, SD difference was 0.19 (95% CI, 0.15 to 0.23) among participants without HBV infection and 0.15 (95% CI, −0.02 to 0.32) among participants with HBV infection (*P* for heterogeneity = .65). For AST, the genetic association differed by HBV status, with a positive genetic association in participants with HBV infection (SD difference, 0.69; 95% CI, 0.11 to 1.28) and a null association in participants without HBV infection (SD difference, −0.01; 95% CI, −0.10 to 0.07) (*P* for heterogeneity = .02).

For chronic liver disease, there was a positive observational association for baseline BMI in women (RR per 1-SD, 1.23; 95% CI, 1.18 to 1.27) and men (RR per 1-SD, 1.06; 95% CI, 1.02 to 1.10), and the associations differed significantly (*P* for heterogeneity < .001) (eFigure 4 and eTable 3 in the Supplement). There was a genetic association of BMI with chronic liver disease in men (RR per 1-SD, 1.89; 95% CI, 1.23 to 2.91) and women (RR per 1-SD, 1.31; 95% CI, 0.88 to 1.96), and the sex differences were not significant (*P* for heterogeneity = .15). For gallbladder disease, observational and genetic associations for BMI did not differ by sex.

The weighted median estimates were concordant with the inverse variance weighted estimates. The Mendelian randomization–Egger estimates were less precise than the inverse variance estimates (eFigure 5 in the Supplement). The intercepts in the Mendelian randomization–Egger regressions were 0.001 (95% CI, −0.02 to 0.02) for chronic liver disease and 0.005 (95% CI, −0.05 to 0.015) for gallbladder disease. The genetic associations persisted when restricting the score to 73 SNVs that did not show different associations with BMI in East Asian populations (eFigure 5 in the Supplement).

### Meta-analysis of CKB and UKB

**Figure 2.**
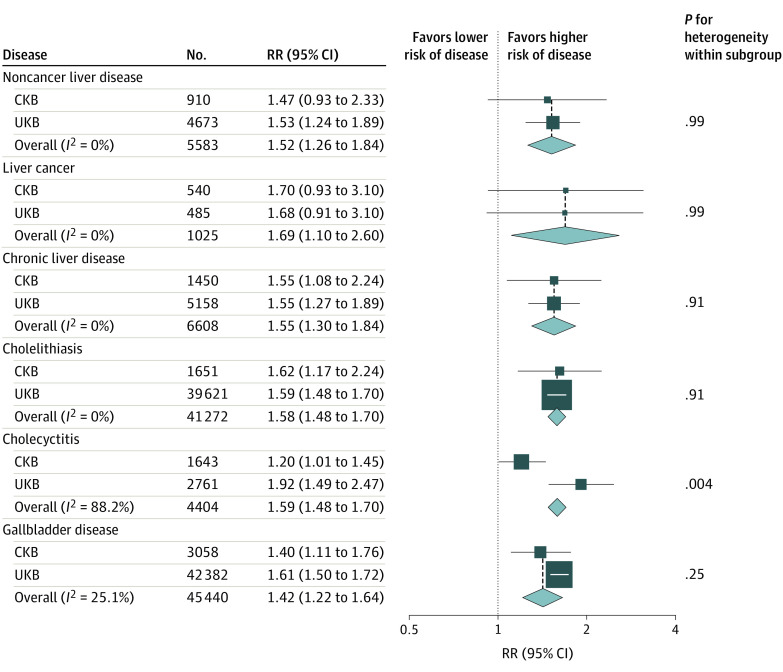
Meta-analysis of the Genetic Associations of Body Mass Index With Hepatobiliary Diseases Boxes indicate the relative risks (RRs) of hepatobiliary diseases associated with 1-SD higher genetically determined body mass index in the China Kadoorie Biobank (CKB) and UK Biobank (UKB) studies, with the size of the box inversely proportional to the variance of the log RR; diamonds, the summary RRs for CKB and UKB; and No., the number of individuals with the disease in the biobank sample.

The UK Biobank (UKB) study found positive genetic associations of BMI with risk of chronic liver disease (RR per 1-SD increase, 1.55; 95% CI, 1.27 to 1.89; *P* for heterogeneity = .95) and gallbladder disease (RR per 1-SD increase, 1.61; 95% CI, 1.50 to 1.72; *P* for heterogeneity = .16), with no heterogeneity between disease subtypes (eTable 4 in the Supplement). In the meta-analysis of CKB and UKB estimates, there were positive genetic associations of BMI with hepatobiliary diseases across subtypes, with little heterogeneity between the 2 studies (Figure 2). Each 1-SD higher genetically determined BMI was associated with a 55% greater risk of chronic liver disease (RR, 1.55; 95% CI, 1.30 to 1.84) and a 42% higher risk of gallbladder disease (RR, 1.42; 95% CI, 1.22 to 1.64).

## Discussion

In this cohort study of a relatively lean Chinese population, there were concordant positive associations of measured BMI and genetically instrumented BMI with hepatobiliary diseases. There were genetic associations of BMI with liver biomarkers, including liver enzymes (ie, ALT and γ-glutamyl transferase), as well as steatosis and fibrosis scores, consistent with the observational associations. The genetic associations of BMI with liver diseases and biomarkers were consistent regardless of HBsAg status. The genetic associations of BMI with hepatobiliary diseases in the CKB study were also consistent with the findings in the UKB study and a previous Mendelian randomization study in the European population,^13,14^ suggesting that general adiposity may be a risk factor for hepatobiliary diseases in relatively lean Chinese adults regardless of the underlying disease causes.

To date, 1 study has assessed the genetic associations of BMI with hepatobiliary diseases, as reported in separate 2013 and 2019 publications,^13,14^ with findings consistent with this study. The 2019 study involved approximately 100 000 participants aged 20 years and older from the Danish general population, including 616 individuals with chronic liver disease (approximately 60% with alcohol-related disease) and 4106 individuals with gallbladder disease (*ICD-10* K80 and K81) studied over a period of 30 years. The mean (SD) BMI (26 [4.3]) was higher than in the CKB study (23.8 [3.4]). Similar to what was done in the CKB and UKB studies, the 2019 study ascertained hepatobiliary diseases through linkages to death registries and hospital records. For BMI instrument, the 2019 study selected 5 SNVs for chronic liver disease and 3 SNVs for gallbladder disease. For chronic liver disease, the authors reported an RR of 1.13 (95% CI, 1.05 to 1.22) per 1-SD greater observational BMI and 2.36 (95% CI, 0.71 to 7.86) per 1-SD greater genetically instrumented BMI.

Although the point estimates for the genetic associations were more extreme than the estimates in the CKB or UKB studies, the RRs had wide CIs owing to the small number of patients studied. In addition, the genetic instrument used in the 2019 Danish study explained half of the BMI variation compared with what the genetic instruments in the CKB and UKB studies explained. For gallbladder disease, Stender et al^13^ reported RRs of 1.34 (95% CI, 1.28 to 1.39) per 1-SD higher observational BMI and 1.96 (95% CI, 0.98 to 3.95) per 1-SD higher genetically instrumented BMI. In the CKB study, the genetic associations of BMI with gallbladder were similar in men and women, as were the observational associations.

In our study’s Chinese population, the genetic associations of BMI with chronic liver disease and gallbladder disease were not significantly different from the observational associations. The associations for the genetically instrumented BMI may reflect the longer duration of adiposity. In addition, the observational estimates could have been affected by residual confounding and reverse causality, particularly for chronic liver disease. Smoking is a major risk factor for cirrhosis and liver cancer and is associated with lower BMI^21,22^ and therefore is a negative confounder between BMI and chronic liver disease.

Approximately 70% of men and less than 3% of women smoked in CKB, so the association of BMI with chronic liver disease may have been confounded by smoking. There was no significant difference between the genetic estimate in men and that in women. In addition, our previous reports in CKB showed that the inverse associations of BMI with liver cancer and cirrhosis attenuated toward the null and lost significance when excluding the first 5 years of follow-up.^12,23^ This finding suggests that participants may have subclinical or undetected diseases at baseline that may affect adiposity at baseline, resulting in reverse causality.

For the observational estimates, the associations for BMI differed by chronic liver disease subtypes. This is likely because reverse causality disproportionally affects liver cancer and cirrhosis (accounting for 65% of the total chronic liver disease population in CKB) and these subtypes have latency periods of more than 10 years.^24,25^ When restricting the analysis to individuals who were never regular smokers or when excluding the first 5 years of follow-up, the inverse associations of BMI with risk of cirrhosis and liver cancer in the BMI range 15 to 24 were not significant and there were positive associations in the BMI range 24 to 50 (eFigure 6 in the Supplement).

Obesity leads to increased fat deposition within the hepatocytes and to the development of hepatic steatosis.^26^ In addition, obesity is associated with higher levels of oxidative stress and inflammation and subsequent liver fibrosis, cirrhosis, and liver cancer.^26^ In populations in the West, where nonalcoholic fatty liver disease is the major cause of liver cancer, obesity plays an important role in causing liver cancer.^24^ However, HBV is the major cause of liver cancer in East Asia, where up to 40% of individuals with HBV-related liver cancer do not have underlying cirrhosis; among these individuals, cancer is caused by integration of the HBV genome into the host’s intracellular DNA.^27^

Although fatty liver index has been used as a proxy for liver fat, this approach has not been validated among patients with HBV. Nonetheless, a 2007 in vivo study^28^ showed that chronic HBV infection may have a synergistic effect with obesity on hepatic lipid accumulation and the development of steatosis, and these effects may confer higher risks of cirrhosis and liver cancer. Future studies are warranted to develop markers of liver fat among individuals with HBV infection and to assess the genetic association of BMI with liver fat.

Approximately 90% of gallstones are cholesterol stones in Western populations, whereas the proportion is approximately 80% in Chinese populations.^29,30^ In individuals with obesity, the activity of 3-hydroxy-3-methyl-glutaryl-coenzyme A reductase is upregulated and cholesterol is hypersecreted from the liver into the bile.^31,32^ The high rate of cholesterol secretion is associated with supersaturation of cholesterol in the bile and disruption of the normal function of the gallbladder.^33^ In addition, individuals with obesity have higher gallbladder volumes and lower gallbladder motility, conditions associated with higher risks of gallstones.^34,35^

The strengths of the CKB study included the prospective design, large and diverse study population, detailed adjustment for risk factors for hepatobiliary diseases, validity of the genetic scores developed for BMI, and ascertainment of hepatobiliary diseases through linkage to hospital records in addition to death and cancer registries.

### Limitations

Our study had several limitations. First, it is plausible that a subset of SNVs in the BMI genetic score affect hepatobiliary diseases independently of BMI, potentially violating the assumptions of Mendelian randomization. However, we showed that Mendelian randomization–Egger estimates and weighted median estimates were broadly consistent with the inverse variance weighted estimates in CKB. In addition, our findings are generally concordant with previous Mendelian randomization studies conducted in Denmark using different genetic variants to construct the BMI genetic score. Second, gallbladder disease and nonalcoholic fatty liver disease were primarily ascertained through linkage to medical records, which might lead to underdiagnosis. However, our results are probably valid because: (1) of all nonalcoholic fatty liver disease diagnoses between 2013 and 2015, we found that over 90% of individuals hospitalized for the disease were diagnosed by ultrasound or computed tomography; (2) our risk estimates agreed with previous studies of ultrasound-detected nonalcoholic fatty liver disease and symptomatic gallstone disease; and (3) hospitalization for nonalcoholic fatty liver disease has been shown to be a valid diagnosis in previous CKB reports^12,36^ on adiposity and diabetes. We defined a suspected nonalcoholic fatty liver disease case as 1 in which the individual had elevated ALT levels (≥33 U/L for men and ≥25 for women; to convert to microkatal per liter, multiply by 0.0167) in approximately 18 000 participants with clinical biochemistry data and negative HBsAg test results, finding a similar genetic estimate for elevated ALT and hospitalized nonalcoholic fatty liver disease (eMethods in the Supplement). Third, BMI is a marker of general adiposity, but central adiposity is also a risk factor associated with hepatobiliary diseases. Future studies are warranted in Chinese populations to assess the genetic associations of central adiposity with hepatobiliary diseases.

## Conclusions

Genetically instrumented BMI was associated with higher risks of hepatobiliary diseases in this cohort study, and consistent associations were observed across disease subtypes. For liver biomarkers, there were genetic associations of BMI with liver enzymes (ie, ALT and γ-glutamyl transferase), as well as steatosis and fibrosis scores. For liver diseases and biomarkers, the genetic associations for BMI were consistent among participants regardless of HBV status. These findings suggest that adiposity may be a risk factor associated with hepatobiliary diseases in the Chinese population regardless of the underlying disease cause. The findings additionally provide support for lifestyle interventions, including dietary intervention and weight loss, to prevent hepatobiliary diseases in the general population.

## Notes

Supplement. **eAppendix.** Supplementary Methods **eTable 1.** ICD-10 Code for Hepatobiliary Diseases in CKB **eTable 2.** Genetic Variants Associated With BMI in the GIANT Consortium **eTable 3.** Observational and Genetic Associations of BMI With Risk of Hepatobiliary Diseases by Sex **eTable 4.** Causal Associations of BMI With Risk of Hepatobiliary Diseases in UKB **eFigure 1.** Flow Diagram **eFigure 2.** Associations of Potential Confounders With BMI Genetic Score **eFigure 3.** Observational Associations of BMI With Risk of Hepatobiliary Diseases **eFigure 4.** Observational and Genetic Associations of BMI With Risk of Hepatobiliary Diseases by Sex **eFigure 5.** Sensitivity Analysis **eFigure 6.** Observational Associations of BMI With Risk of Hepatobiliary Diseases With Different Exclusions
