# Supplementary material for: Observational and Genetic Associations of Body Mass Index and Hepatobiliary Diseases in a Relatively Lean Chinese Population
Source: JAMA Netw Open. 2020 Oct 2;3(10):e2018721. doi: 10.1001/jamanetworkopen.2020.18721 (PMC7532388; doi:10.1001/jamanetworkopen.2020.18721)
Supplement: Supplement. — eAppendix. Supplementary Methods eTable 1. ICD-10 Code for Hepatobiliary Diseases in CKB eTable 2. Genetic Variants Associated With BMI in the GIANT Consortium eTable 3. Observational and Genetic Associations of BMI With Risk of Hepatobiliary Diseases by Sex eTable 4. Causal Associations of BMI With Risk of Hepatobiliary Diseases in UKB eFigure 1. Flow Diagram eFigure 2. Associations of Potential Confounders With BMI Genetic Score eFigure 3. Observational Associations of BMI With Risk of Hepatobiliary Diseases eFigure 4. Observational and Genetic Associations of BMI With Risk of Hepatobiliary Diseases by Sex eFigure 5. Sensitivity Analysis eFigure 6. Observational Associations of BMI With Risk of Hepatobiliary Diseases With Different Exclusions [file jamanetwopen-e2018721-s001.pdf]

Supplementary Online Content

Pang Y, Kartsonaki C, Lv J, et al. Observational and genetic associations of body mass index and hepatobiliary diseases in a relative lean Chinese population. *JAMA Netw Open*. 2020;3(10):e2018721. doi:10.1001/jamanetworkopen.2020.18721

eAppendix. Supplementary Methods

eTable 1. ICD-10 Code for Hepatobiliary Diseases in CKB

eTable 2. Genetic Variants Associated With BMI in the GIANT Consortium

eTable 3. Observational and Genetic Associations of BMI With Risk of Hepatobiliary Diseases by Sex

eTable 4. Causal Associations of BMI With Risk of Hepatobiliary Diseases in UKB

eFigure 1. Flow Diagram

eFigure 2. Associations of Potential Confounders With BMI Genetic Score

eFigure 3. Observational Associations of BMI With Risk of Hepatobiliary Diseases

eFigure 4. Observational and Genetic Associations of BMI With Risk of Hepatobiliary Diseases by Sex

eFigure 5. Sensitivity Analysis

eFigure 6. Observational Associations of BMI With Risk of Hepatobiliary Diseases With Different Exclusions

This supplementary material has been provided by the authors to give readers additional information about their work.

© 2020 Pang Y et al. *JAMA Network Open.*

eAppendix. Supplementary Methods

Assessment of random plasma glucose and HBsAg

At the baseline survey, a 10-ml nonfasting (with the time since the participant last ate recorded) blood sample was collected from participants into an ethylene diamine tetraacetic acid vacutainer (EDTA) vacutainer (BD Hemogard, NJ, US). A small sample of this was used for on-site rapid dipstick testing of random plasma glucose and hepatitis B antigen (HBsAg). HBsAg was measured using the ACON dipstick (ACON Biotech, CA, US). RPG level was measured using the SureStep Plus System (Johnson & Johnson, CA, US), regularly calibrated with manufacturer quality control solution.[1,2]

Quality control

In the baseline survey, standardised procedures were used at all 10 study sites and thorough quality control measures were undertaken. A regional coordinating centre and survey team were established in each of the 10 study sites involving 15 full-time staff with medical qualifications and fieldwork experience. To standardise procedures for the study management, field survey, and collection and validation of long-term follow-up data, a range of Standard Operating Procedures (SOPs) were developed and were used across 10 sites.

Within several weeks of the initial baseline survey in a particular community, a quality control (QC) survey was done. The QC survey involved approximately 3% of the participants randomly selected from that community and repeat questionnaire and measures on selected items were collected. 15,728 individuals had available QC survey data, with the mean length of time between baseline and QC survey being 17 days. For most of the variables examined, there was good agreement between the baseline and QC data, particularly for physical measurements.

© 2020 Pang Y et al. *JAMA Network Open.*

The QC survey data showed good agreement between baseline and repeat measures of adiposity measures, with extremely high correlations for height, weight and BMI (Spearman correlation coefficient: 0.99, 0.96 and 0.93, respectively).[1]

Assessment of liver biomarkers

17 biomarkers were measured by standard clinical biochemistry assays in 18,181 participants from a nested case-control study of stroke and CHD (5486 cases of IS, 5067 of ICH, 1008 of MI, 277 of fatal ischaemic heart disease, 6343 controls; all free of prior vascular disease and cancer, and not on statin therapy), at the Wolfson Laboratory, CTSU, University of Oxford, UK. The biochemistry measurements included the liver enzymes alanine aminotransferase (ALT), aspartate transaminase (AST), and gamma-glutamyl transferase (GGT), and triglycerides (TGs) used to assess liver steatosis. Liver function was measured by ALT, AST, and GGT. Steatosis was measured by the fatty liver index (FLI) using the following formula:[3]

$$e^{0.953\times log_e TG+0.139\times BMI+0.718\times log_e GGT+0.052\times WC-15.745} / (1+e^{0.953\times log_e TG+0.139\times BMI+0.718\times log_e GGT+0.052\times WC-15.745}) \times 100.$$

Fibrosis was measured by the BARD score, calculated as the weighted sum of BMI >28 (1 point), AST/ALT ratio >0.8 (2 points), and diabetes (1 point).[4] The FLI is a non-invasive diagnostic biomarker for NAFLD and provides a quantitative assessment of steatosis.[3] The BARD score is a non-invasive model to detect liver fibrosis caused by various aetiologies and has been shown to predict advanced fibrosis with good sensitivity and specificity.[4]

Genotyping

Genotyping was conducted using a custom-designed 800K-SNP array (Axiom; Affymetrix) with imputation to 1000 Genomes Phase 3. For this study, Genotype data were available for samples from 100,408 participants passing QC (overall call rate >99.97% across all variants).

© 2020 Pang Y et al. *JAMA Network Open.*

This included a population-based sample of 75,736 participants randomly selected from the total CKB cohort. The remaining 24,672 participants were genotyped as part of nested case-control studies of incident stroke, coronary heart disease (CHD), or chronic obstructive pulmonary disease. To avoid potential ascertainment bias, only the 75,736 population-representative subset of participants were used for genetic analyses of hepatobiliary outcomes. All participants with clinical biochemistry measures were genotyped.

Statistical analysis for liver biomarkers

In the analysis of BMI and liver biomarkers, participants with prior cancer or liver disease were excluded from the analysis, leaving 18,053 participants for the observational analysis. In both the observational and genetic analyses, inverse probability of sampling weights (i.e. inclusion in the nested case-control study) were developed to ensure that our analysis accounted for the inclusion/exclusion criteria and sampling scheme for the nested case-control study.[5] Cases and controls were assigned different weights to reflect the different proportions of cases and controls from eligible participants in the entire CKB cohort. The weights were calculated separately for controls and cases as the number of eligible participants divided by the number selected in the nested case-control study. The weights were 307.35 for controls, 4.47 for CHD cases, 27.82 for IS cases, and 6.78 for ICH cases. All liver biomarkers were standardised to have a standard deviation (SD) of 1 (except for the BARD score). In observational analysis, linear regression was used to assess the associations of BMI with liver markers. In Mendelian randomisation analysis, the genetic associations of BMI with liver biomarkers were estimated by the two-stage least squares estimator method using individual participant-level data (IPD). In the first stage, the associations between BMI genetic score and BMI were examined in 75,736 participants in

© 2020 Pang Y et al. *JAMA Network Open.*

the GWAS population subset using linear regression adjusting for age, age squared, sex, 10 regions, the first 12 principal components, education, smoking, alcohol, and HBsAg. In the second stage, the associations of the resulting predicted values with liver biomarkers were examined using linear regression adjusting for the same covariates. There were 17,567 participants in the second stage had available genotype and liver biomarkers data.

Meta-analysis of CKB with UKB

The genetic associations of BMI on hepatobiliary diseases in UKB were calculated using two-sample Mendelian randomisation. Summary statistics of 97 BMI-associated SNPs were retrieved from GIANT.[6] For hepatobiliary diseases in UKB, SNP-outcome effects were obtained from a set of publicly available summary statistics reported by Zhou et al.[7] We used a conventional IVW Mendelian randomisation analysis in which the SNP to disease estimate was regressed on the SNP to BMI using logistic regression, with the y-axis intercept forced through the origin. For each disease, a combined causal estimate was calculated from the causal estimate from each BMI SNP using a random effects meta-analysis. Meta-analyses of the genetic estimates per 1-SD genetically elevated BMI in CKB and UKB yielded pooled estimates for CLD and GBD.

Subgroup and sensitivity analyses

We conducted several subgroup and sensitivity analyses. First, the genetic associations of liver diseases and biomarkers were conducted by HBV infections. Participants were classified as HBV positive if they had a positive HBsAg test at the baseline survey. Second, sex-specific analyses of the observational and genetic associations were conducted as previous studies

© 2020 Pang Y et al. *JAMA Network Open.*

have suggested that the associations of BMI with hepatobiliary diseases differ by sex.[8,9] Third, the genetic associations of BMI with hepatobiliary diseases were conducted using 73 SNPs that did not show different associations with BMI between European and East Asians populations (*p*-value for heterogeneity<0.05).[5] Fourth, we used the MR-Egger and weighted median methods to explore whether findings in CKB depend on the assumption that all the variants have no horizontal pleiotropic effects (i.e. the effects of BMI variants on multiple biological pathways)[10]. MR-Egger method is a statistical approach that allows one or more genetic variants to have pleiotropic effects, while weighted median estimator can give valid estimates even in the presence of horizontal pleiotropy as long as at least half the genetic variants have no pleiotropic effects. Weighted median estimator leads to greater precision in the estimates than MR-Egger (owing to a power penalty)[10].

References

1. Chen Z, Chen J, Collins R, Guo Y, Peto R, Wu F, et al. China Kadoorie Biobank of 0.5 million people: survey methods, baseline characteristics and long-term follow-up. *Int J Epidemiol.* 2011;40(6):1652-1666.
2. Chen Z, Lee L, Chen J, Collins R, Wu F, et al. Cohort profile: the Kadoorie Study of Chronic Disease in China (KSCDC). *Int J Epidemiol.* 2005;34(6):1243-1249.
3. Bedogni G, Bellentani S, Miglioli L, Masutti F, Passalacqua M, Castiglione A, et al. The Fatty Liver Index: a simple and accurate predictor of hepatic steatosis in the general population. *BMC Gastroenterol.* 2006;6:33. doi:10.1186/1471-230X-6-33.
4. Xiao G, Zhu S, Xiao X, Yan L, Yang J, and Wu G. Comparison of laboratory tests, ultrasound, or magnetic resonance elastography to detect fibrosis in patients with nonalcoholic fatty liver disease: A meta-analysis. *Hepatology.* 2017;66(5):1486-1501.
5. Pang Y, Kartsonaki C, Du H, Millwood IY, Guo Y, Chen Y, et al. Physical activity, sedentary leisure time, circulating metabolic markers, and risk of major vascular diseases. *Circ Genom Precis Med*. 2019;12(9):386-396.
6. Locke AE, Kahali B, Berndt SI, Justice AE, Pers TH, Day FR, et al. Genetic studies of body mass index yield new insights for obesity biology. *Nature.* 2015;518(7538):197-206.
7. Zhou W, Nielsen JB, Fritsche LG, Dey R, Gabrielsen ME, Wolford BN, et al. Efficiently controlling for case-control imbalance and sample relatedness in large-scale genetic association studies. *Nat Genet.* 2018;50(9):1335-1341.

© 2020 Pang Y et al. *JAMA Network Open.*

8. Stender S, Nordestgaard BG, Tybjaerg-Hansen A. Elevated body mass index as a causal risk factor for symptomatic gallstone disease: a Mendelian randomization study. *Hepatology.* 2013;58(6):2133-2141.
9. Carter AR, Borges MC, Benn M, Tybjaerg-Hansen A, Davey Smith G, Nordestgaard BG, et al. Combined association of body mass index and alcohol consumption with biomarkers for liver injury and incidence of liver disease: a Mendelian randomization study. *JAMA Netw Open.* 2019;2(3):e190305. doi:10.1001/ja,anetworkopen.2019.0305.
10. Holmes MV, Ala-Korpela M, Smith GD. Mendelian randomization in cardiometabolic disease: challenges in evaluating causality. *Nat Rev Cardiol.* 2017;14(10):577-590.

© 2020 Pang Y et al. *JAMA Network Open.*

eTable 1. ICD-10 code for hepatobiliary diseases in CKB

Description	ICD-10	No. cases whole cohort	No. cases with genetic data
Chronic liver disease			
Alcoholic liver disease	K70	303	65
Cirrhosis	K74	1334	492
Nonalcoholic fatty liver disease	K76.0	2500	275
Viral hepatitis	B18-B19	1776	335
Primary liver cancer	C22	2970	607
Total		7400	1307
Gallbladder disease			
Cholelithiasis	K80	11,101	2193
Cholecystitis	K81	10,291	2068
Total		19,490	3883

© 2020 Pang Y et al. *JAMA Network Open.*

eTable 2. Genetic variants associated with BMI in the GIANT consortium

SNP	BMI-increasing allele	Other allele		EAF		Beta coefficient
				GIANT	CKB	
rs1000940	G	A		0.32	0.38	-0.003
rs10132280	C	A		0.68	0.91	0.026
rs1016287[a]	T	C		0.29	0.21	-0.005
rs10182181	G	A		0.46	0.44	0.035
rs10733682	A	G		0.48	0.52	0.019
rs10938397	G	A		0.43	0.30	0.038
rs10968576	G	A		0.32	0.21	0.015
rs11030104	A	G		0.79	0.52	0.038
rs11057405	G	A		0.90	0.999	0.065
rs11126666	A	G		0.28	0.29	0.013
rs11165643[a]	T	C		0.58	0.77	0.008
rs11191560	C	T		0.09	0.28	0.034
rs11583200	C	T		0.40	0.60	0.003
rs1167827	G	A		0.55	0.93	0.016
rs11688816	G	A		0.52	0.69	0.001
rs11727676	T	C		0.91	0.99	0.053
rs11847697	T	C		0.04	0.001	0.152
rs12016871[a]	T	C		0.20	Not available	–
rs12286929	G	A		0.52	0.27	0.010
rs12401738	A	G		0.35	0.01	0.026
rs12429545	A	G		0.13	0.25	0.035
rs12446632	G	A		0.87	0.9997	0.124
rs12566985	G	A		0.45	0.16	0.041
rs12885454[a]	C	A		0.64	0.56	0.014
rs12940622	G	A		0.57	0.70	0.020
rs13021737	G	A		0.83	0.91	0.085
rs13078960	G	T		0.20	0.005	0.012
rs13107325	T	C		0.07	Rare (<0.01%)	–
rs13191362	A	G		0.88	0.99	0.014
rs13201877	G	A		0.14	0.06	0.006
rs1441264*	A	G		0.61	0.59	0.007
rs1460676	C	T		0.17	0.38	0.005
rs1516725	C	T		0.87	0.95	0.052
rs1528435	T	C		0.63	0.68	0.012
rs1558902	A	T		0.42	0.12	0.099
rs16851483[a]	T	G		0.07	0.24	0.026
rs16907751	C	T		0.92	0.81	0.018
rs16951275	T	C		0.78	0.60	0.034
rs17001654	G	C		0.15	0.02	0.0004
rs17024393	C	T		0.04	Rare (<0.01%)	–

© 2020 Pang Y et al. *JAMA Network Open.*

SNP	BMI-increasing allele	Other allele		EAF		Beta coefficient
				CKB	UKB	
rs17094222	C	T		0.21	0.33	0.017
rs17203016	G	A		0.20	0.16	0.018
rs17405819	T	C		0.70	0.55	0.024
rs17724992	A	G		0.75	0.52	0.021
rs1808579[a]	C	T		0.53	0.47	-0.005
rs1928295	T	C		0.55	0.53	0.012
rs2033529[a]	G	A		0.29	0.19	0.006
rs2033732[a]	C	T		0.75	0.62	0.008
rs205262	G	A		0.27	0.14	0.009
rs2075650	A	G		0.85	0.90	0.002
rs2080454	C	A		0.41	0.51	-0.007
rs2112347	T	G		0.63	0.43	0.020
rs2121279	T	C		0.15	Rare (<0.01%)	–
rs2176040	A	G		0.37	0.07	0.009
rs2176598[a]	T	C		0.25	0.12	0.007
rs2207139[a]	G	A		0.18	0.15	0.036
rs2245368	C	T		0.18	Not available	–
rs2287019	C	T		0.80	0.82	0.024
rs2365389	C	T		0.58	0.13	0.005
rs2650492	A	G		0.30	0.05	0.008
rs2820292[a]	C	A		0.56	0.22	-0.002
rs2836754	C	T		0.61	0.63	0.014
rs29941[a]	G	A		0.67	0.75	0.028
rs3101336	C	T		0.61	0.92	0.024
rs3736485	A	G		0.45	0.16	0.010
rs3810291	A	G		0.67	0.70	0.030
rs3817334	T	C		0.41	0.31	0.020
rs3849570[a]	A	C		0.36	0.49	-0.007
rs3888190	A	C		0.40	0.13	0.015
rs4256980	G	C		0.65	0.62	0.022
rs4740619[a]	T	C		0.54	0.76	0.017
rs4787491	G	A		0.51	0.40	0.015
rs492400[a]	C	T		0.42	0.23	0.001
rs543874[a]	G	A		0.19	0.18	0.058
rs6091540	C	T		0.72	0.70	0.014
rs6465468	T	G		0.30	0.02	0.007
rs6477694	C	T		0.37	0.43	0.014
rs6567160	C	T		0.24	0.20	0.061
rs657452	A	G		0.39	0.25	0.009
rs6804842	G	A		0.57	0.63	0.008
rs7138803	A	G		0.38	0.28	0.023

© 2020 Pang Y et al. *JAMA Network Open.*

rs7141420[a]	T	C		0.53	0.56	0.008
**SNP**	**BMI-increasing allele**	**Other allele**		**EAF**		**Beta coefficient**
				**CKB**	**UKB**	
rs7164727[a]	T	C		0.69	0.73	0.013
rs7239883	G	A		0.39	0.30	0.012
rs7243357	T	G		0.81	0.80	0.031
rs758747	T	C		0.27	0.34	0.019
rs7599312	G	A		0.72	0.97	0.019
rs7715256	G	T		0.42	0.03	0.009
rs7899106	G	A		0.05	0.01	0.066
rs7903146	C	T		0.71	0.96	0.012
rs9374842[a]	T	C		0.75	0.90	0.016
rs9400239	C	T		0.69	0.73	0.018
rs9540493	A	G		0.46	0.26	0.010
rs9641123	C	G		0.43	0.30	-0.004
rs977747	T	G		0.39	0.05	-0.002
rs9914578	G	C		0.21	0.22	0.019
rs9925964	A	G		0.62	0.09	-0.012

[a] SNPs that showed different associations with BMI between European and East Asian populations in GIANT.

© 2020 Pang Y et al. *JAMA Network Open.*

eTable 3. Observational and genetic associations of BMI with risk of hepatobiliary diseases by sex

	Observational			Genetic	
	RR per 1-SD (95% CI)	*p* for heterogeneity		RR per 1-SD (95% CI)	*p* for heterogeneity
**Non-cancer CLD**					
Male	1.11 (1.06, 1.16)	<0.001		1.60 (1.05, 2.43)	0.34
Female	1.34 (1.28, 1.40)			1.20 (0.79, 1.83)	
**Liver cancer**					
Male	0.95 (0.90, 1.01)	0.93		2.02 (1.05, 3.89)	0.79
Female	0.95 (0.89, 1.02)			1.80 (1.00, 3.23)	
**Gallstone disease**					
Male	1.45 (1.39, 1.52)	0.42		1.86 (1.35, 2.57)	0.46
Female	1.42 (1.39, 1.46)			1.57 (1.15, 2.14)	
**Cholecystitis**					
Male	1.17 (1.12, 1.22)	0.84		1.03 (0.60, 1.74)	0.24
Female	1.17 (1.14, 1.20)			1.49 (1.07, 2.08)	

The model was adjusted for age at baseline, age squared, 10 regions, 10 PCs (for GRS), HBsAg (for CLD), education, smoking, alcohol, and total physical activity.

© 2020 Pang Y et al. *JAMA Network Open.*

eTable 4. Causal associations of BMI with risk of hepatobiliary diseases in UKB

Disease	PheCode	Disease description	No. cases	RR (95% CI)	P for heterogeneity
Liver cancer	155	Cancer of liver and intrahepatic bile duct	344	1.52 (0.74, 3.11)	
	155.1	Malignant neoplasm of liver, primary	141	2.22 (0.69, 7.15)	
Non-cancer liver disease	571	Chronic liver disease and cirrhosis	2895	1.48 (1.14, 1.92)	
	571.5	Other chronic nonalcoholic liver disease	1664	1.67 (1.16, 2.42)	
	571.51	Cirrhosis of liver without mention of alcohol	114	1.31 (0.38, 4.48)	
Chronic liver disease			***5158***	***1.55 (1.27, 1.89)***	***0.95***
Gallstones	574	Cholelithiasis and cholecystitis	16,225	1.61 (1.43, 1.80)	
	574.1	Cholelithiasis	13,777	1.57 (1.39, 1.77)	
	574.11	Cholelithiasis with acute cholecystitis	1513	1.42 (1.01, 2.00)	
	574.12	Cholelithiasis with other cholecystitis	5472	1.43 (1.19, 1.71)	
	574.2	Calculus of bile duct	2634	2.08 (1.60, 2.69)	
Cholecystitis	574.3	Cholecystitis without cholelithiasis	2761	1.92 (1.49, 2.47)	
Gallbladder disease			***42,382***	***1.61 (1.50, 1.72)***	***0.16***

In UKB, 5158 CLD and 42,383 GBD had developed over a median of 5 years of follow-up. The main subtype was cirrhosis (2895, 56%) for CLD and cholelithiasis (36,987, 87%) for GBD.

© 2020 Pang Y et al. *JAMA Network Open.*

eFigure 1. Flow diagram

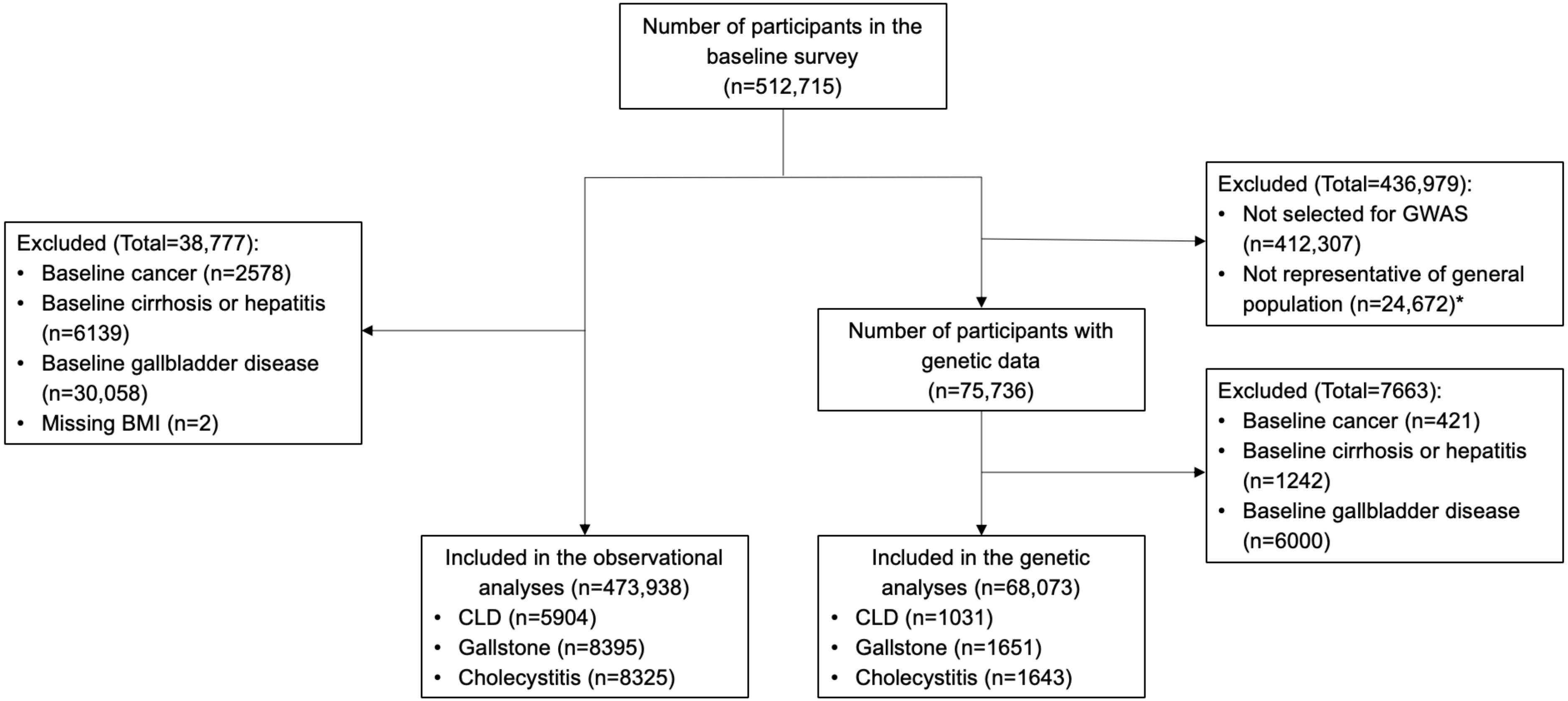

*The participants (n=24,672) were enriched for cases as part of a case-control study.

Abbreviations: CVD, cardiovascular disease; CLD, chronic liver disease.

© 2020 Pang Y et al. *JAMA Network Open.*

eFigure 2. Associations of potential confounders with BMI genetic score

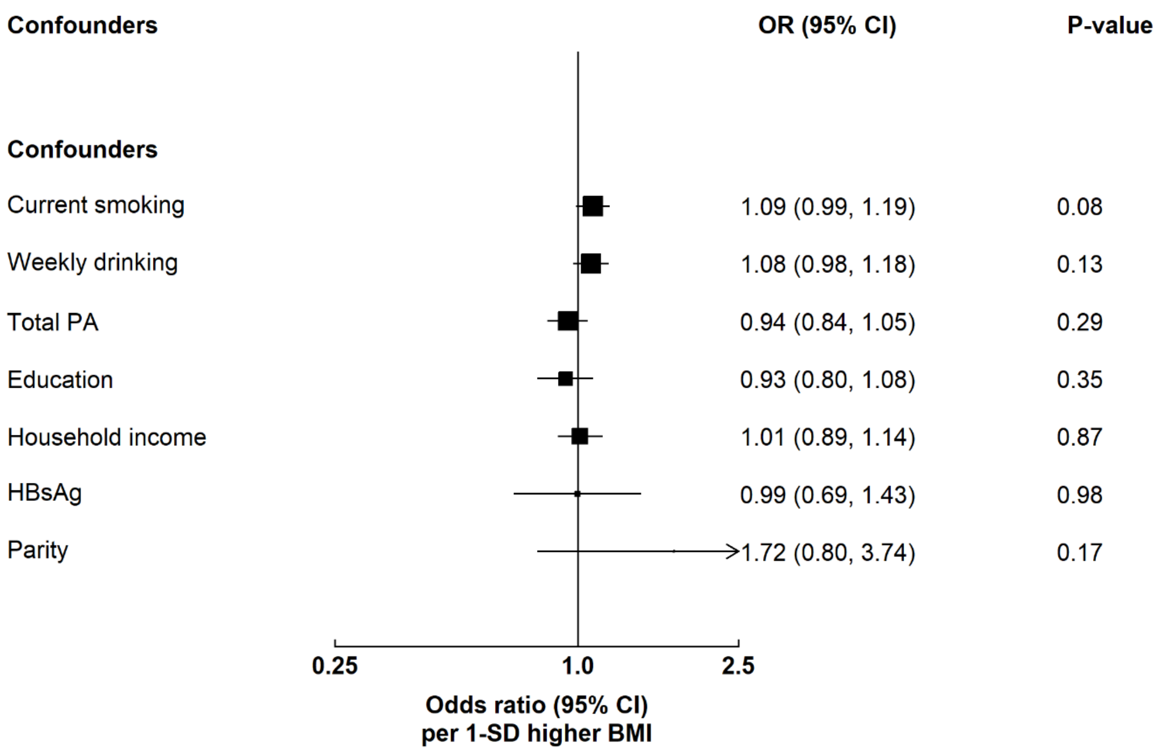

The model was adjusted for age at baseline, age squared, 10 regions, 10 PCs (for GRS), HBsAg (for CLD), education, smoking, alcohol, and total physical activity, where appropriate.

Potential confounders were dichotomised: current smoking (yes vs no), weekly drinking (yes vs no), total PA (≥30 vs <30 MET-h/day), HBsAg (positive vs negative), education (≥9 vs <9 years), household income (≥35,000 vs <35,000 RMB/year), and parity (any number of live births vs nulliparity).

© 2020 Pang Y et al. *JAMA Network Open.*

eFigure 3. Observational associations of BMI with risk of hepatobiliary diseases

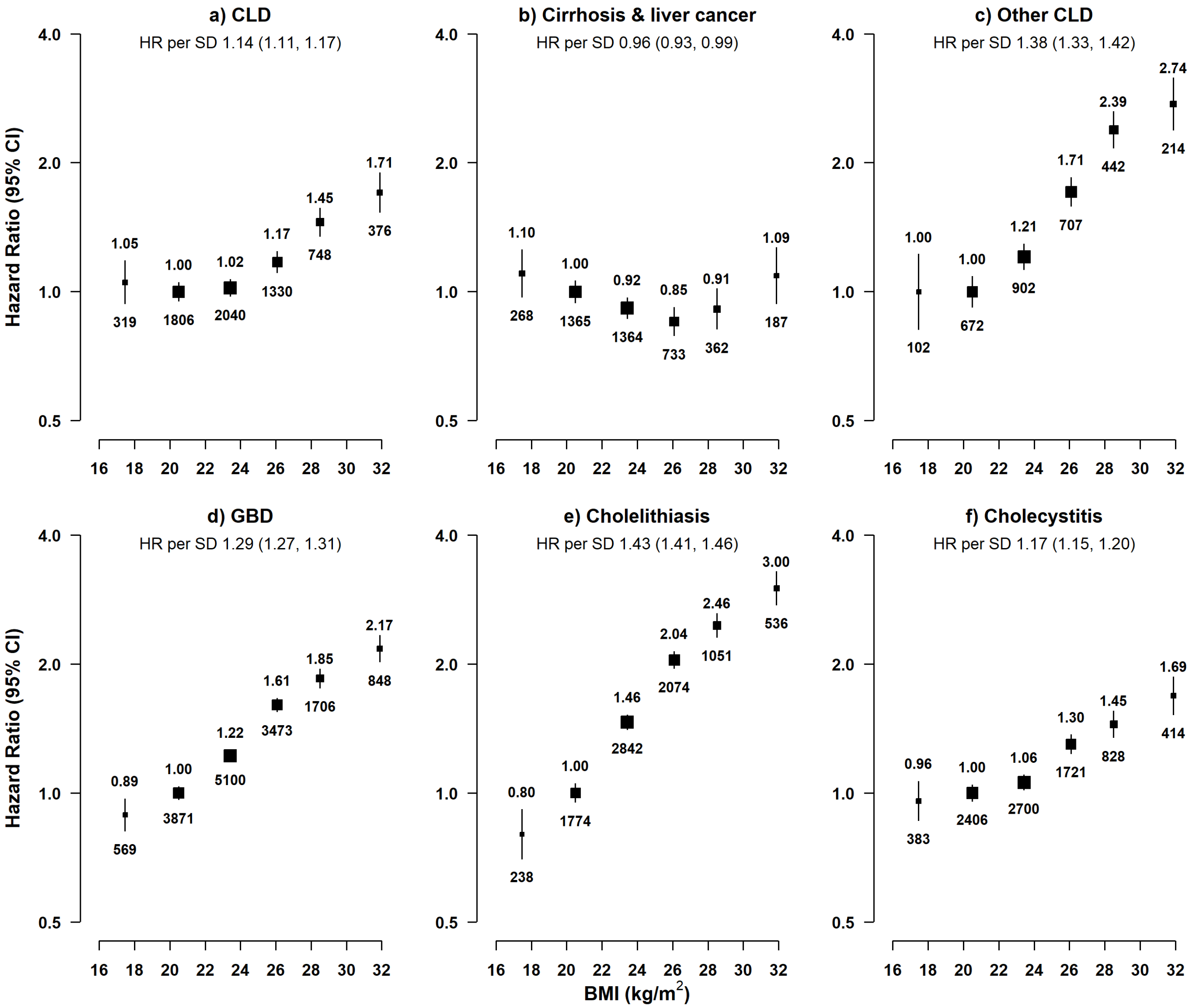

© 2020 Pang Y et al. *JAMA Network Open.*

eFigure 4. Observational and genetic associations of BMI with risk of hepatobiliary diseases by sex

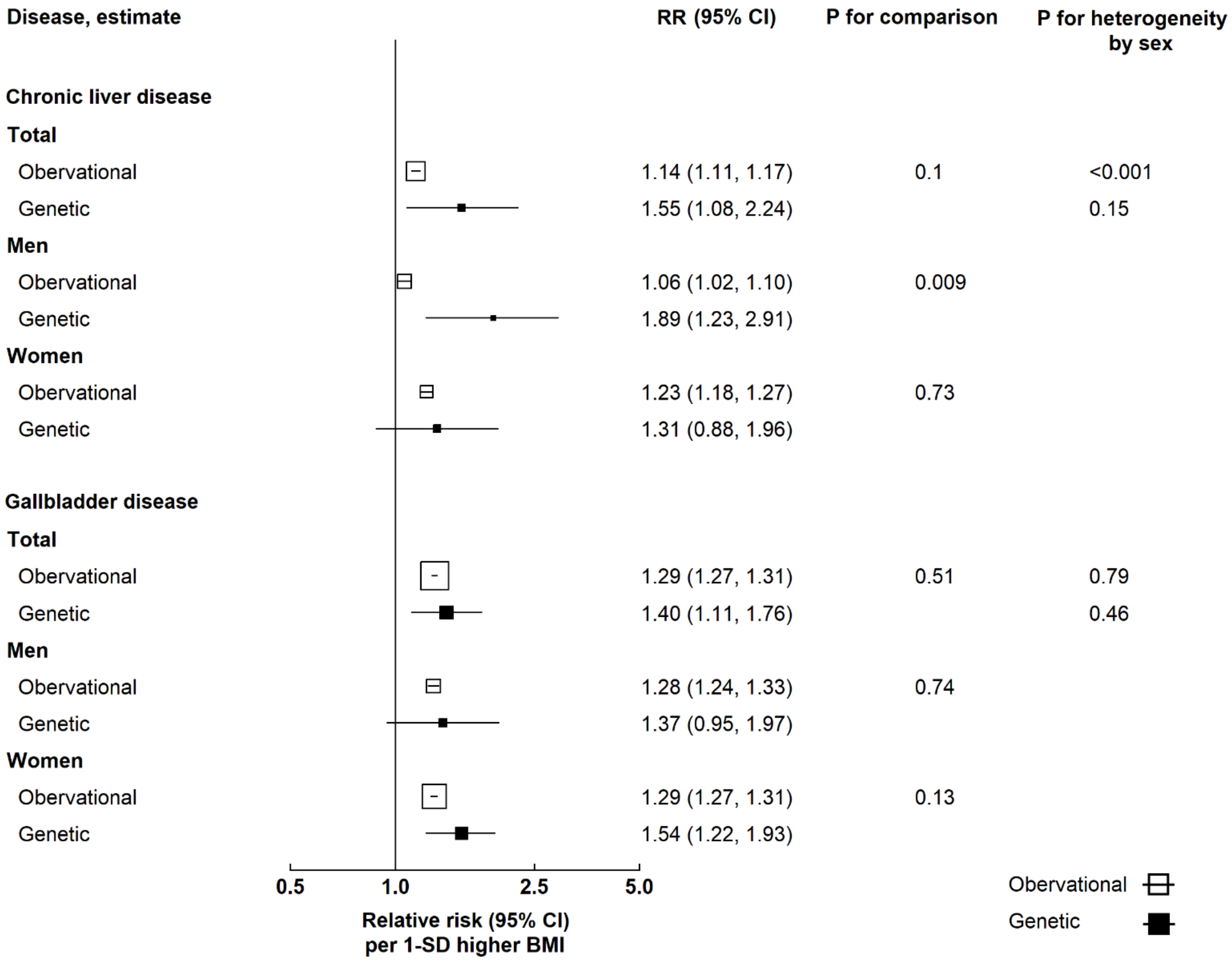

The model was adjusted for age at baseline, age squared, sex, 10 regions, 12 PCs (for GRS), HBsAg (for CLD), education, smoking, alcohol, and total physical activity, where appropriate.

© 2020 Pang Y et al. *JAMA Network Open.*

eFigure 5. Sensitivity analysis

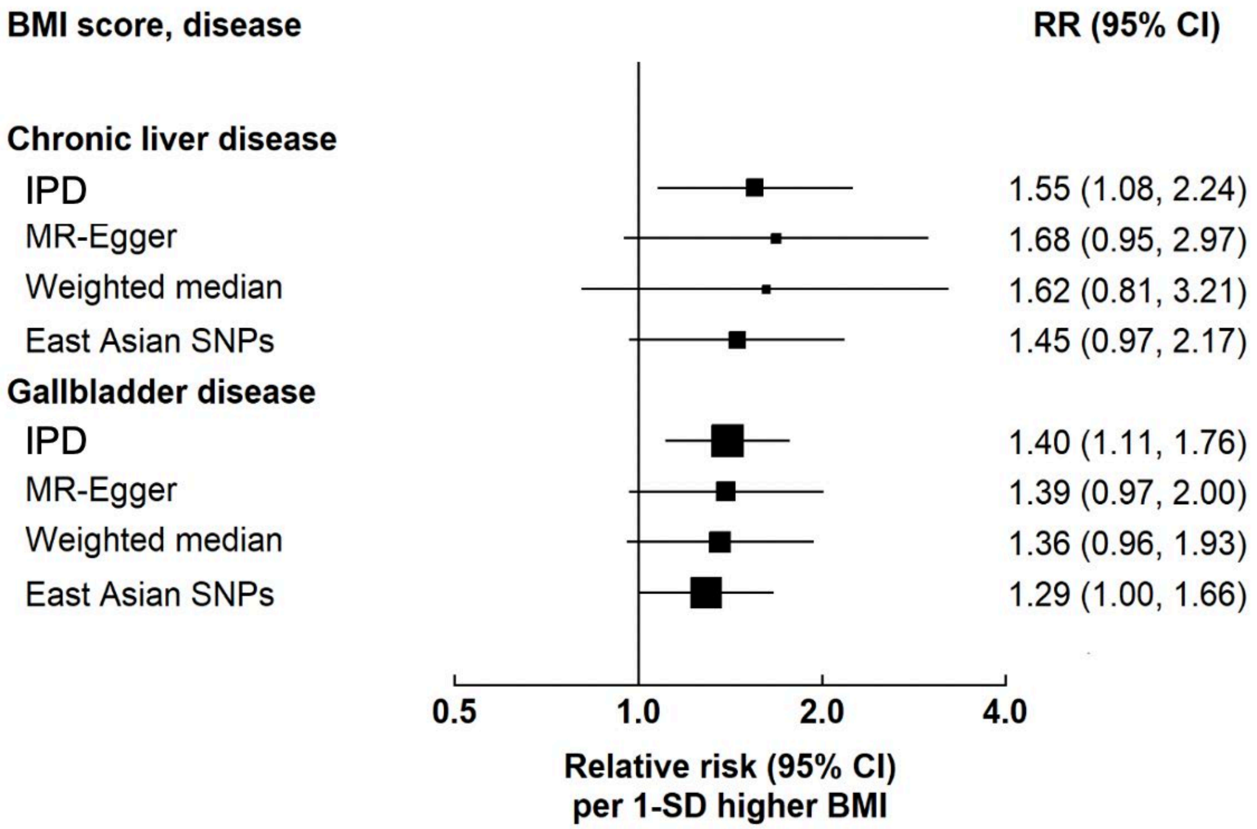

Two-sample MR estimates: chronic liver disease 1.58 (1.12-2.23), gallbladder disease 1.44 (1.16-1.79).

The associations of genetically-predicted BMI with hepatobiliary diseases were also assessed by two-sample Mendelian randomisation using summary statistics from the GIANT (i.e. SNP-BMI) together with summary-specific estimates in CKB (i.e. SNP-disease). The derivation of the summary estimates in CKB used the same adjustment as the individual participant data (IPD) analysis. Inverse-variance weighted (IVW) analysis was performed by linear regression of the SNP-disease associations on the SNP-BMI associations.

© 2020 Pang Y et al. *JAMA Network Open.*

eFigure 6. Observational associations of BMI with risk of hepatobiliary diseases with different exclusions

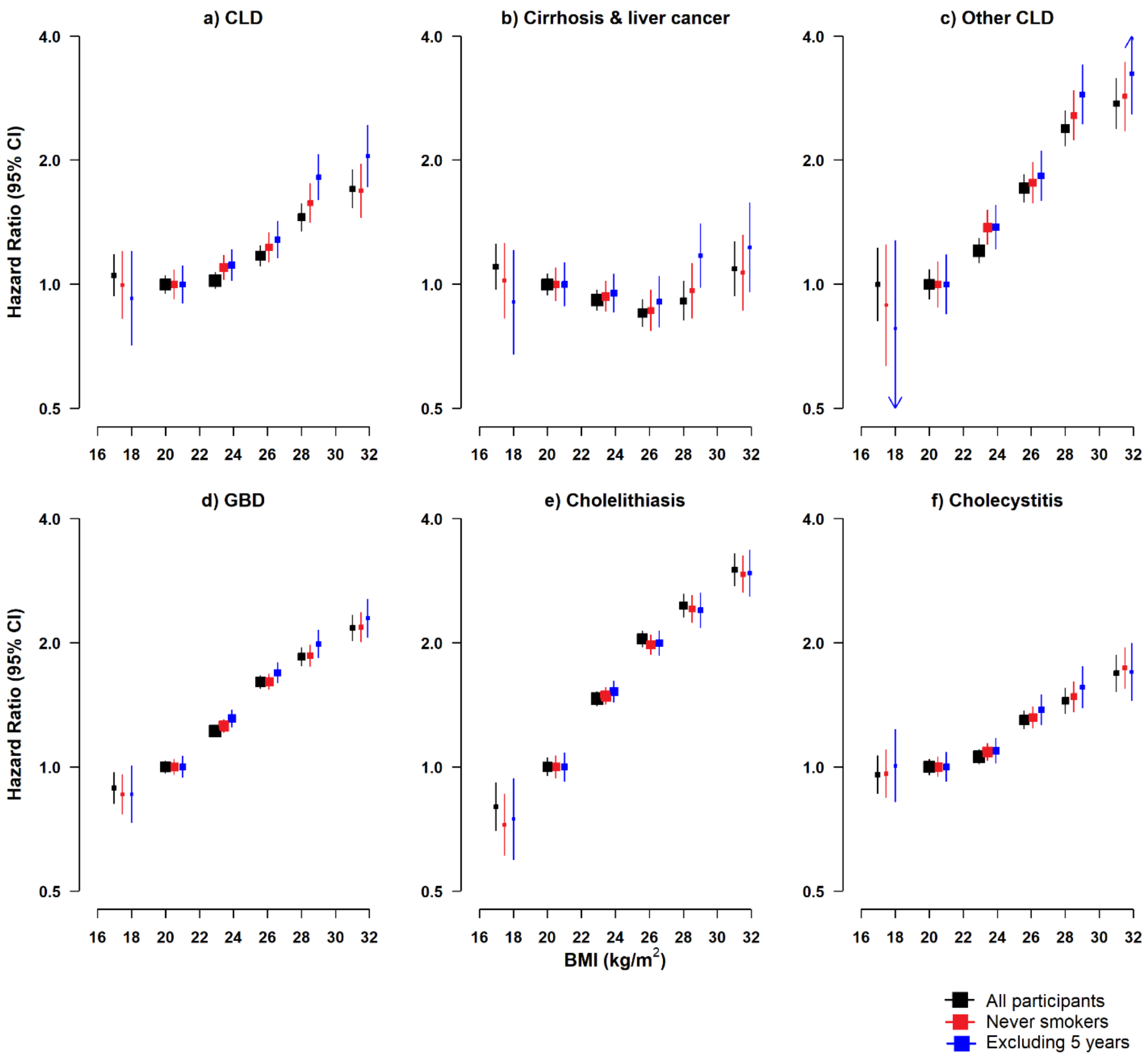

© 2020 Pang Y et al. *JAMA Network Open.*
